# National-scale cropland mapping based on spectral-temporal features and outdated land cover information

**DOI:** 10.1371/journal.pone.0181911

**Published:** 2017-08-17

**Authors:** François Waldner, Matthew C. Hansen, Peter V. Potapov, Fabian Löw, Terence Newby, Stefanus Ferreira, Pierre Defourny

**Affiliations:** 1 Université catholique de Louvain, Earth and Life Institute-Environmental Sciences, 2 Croix du Sud, 1348 Louvain-la-Neuve, Belgium; 2 Department of Geographical Sciences, University of Maryland, 4321 Hartwick Road, College Park, Maryland, United States of America; 3 MapTailor Geospatial Consulting GbR, 53113 Bonn, Germany; 4 Agricultural Research Council, Private Bag X79, 0001 Pretoria, South Africa; 5 GeoTerra Image, 295 Persequor Park, 0020 Pretoria, South Africa; University of Maryland at College Park, UNITED STATES

## Abstract

The lack of sufficient ground truth data has always constrained supervised learning, thereby hindering the generation of up-to-date satellite-derived thematic maps. This is all the more true for those applications requiring frequent updates over large areas such as cropland mapping. Therefore, we present a method enabling the automated production of spatially consistent cropland maps at the national scale, based on spectral-temporal features and outdated land cover information. Following an unsupervised approach, this method extracts reliable calibration pixels based on their labels in the outdated map and their spectral signatures. To ensure spatial consistency and coherence in the map, we first propose to generate seamless input images by normalizing the time series and deriving spectral-temporal features that target salient cropland characteristics. Second, we reduce the spatial variability of the class signatures by stratifying the country and by classifying each stratum independently. Finally, we remove speckle with a weighted majority filter accounting for per-pixel classification confidence. Capitalizing on a wall-to-wall validation data set, the method was tested in South Africa using a 16-year old land cover map and multi-sensor Landsat time series. The overall accuracy of the resulting cropland map reached 92%. A spatially explicit validation revealed large variations across the country and suggests that intensive grain-growing areas were better characterized than smallholder farming systems. Informative features in the classification process vary from one stratum to another but features targeting the minimum of vegetation as well as short-wave infrared features were consistently important throughout the country. Overall, the approach showed potential for routinely delivering consistent cropland maps over large areas as required for operational crop monitoring.

## Introduction

South African households’ vulnerability to hunger has declined in the past ten years from 24% to 12% in 2011 [1, 2]. In 2013, 2.8 million households –comprising 11 million people– were deemed food insecure [3]. The measures and programs initiated by the South African government appear beneficial even though they could be run more effectively [4], and particularly the lack of access to land must be addressed through sustainable, income-independent measures, such as the promotion of subsistence farming. Besides, progress in achieving food security is in jeopardy as the agriculture sector faces considerable impact from climate change. South Africa, on average, has been hotter and drier during the last 10 years than during the 1970s. Those changes in climate and water use affect the livelihoods of the vast majority of people, especially those already considered vulnerable [3]. [5] employed an econometric model to estimate how sensitive the nation’s agriculture may be to changes in rainfall. For the country as a whole, they concluded that each 1% decline in rainfall is likely to lead to a 1.1% decline in the production of maize and a 0.5% decline in production of winter wheat. Reducing risk through raising awareness as well as strengthening early warning systems and warning dissemination helps to build resilient farming communities. Therefore, the Department of Agriculture, Forestry and Fisheries of South Africa has developed and implemented an Early Warning System disseminating extreme weather warnings [3].

Up-to-date and dependable satellite-derived cropland maps are one crucial element of crop monitoring and early warning systems because they allow subsequent analyses such as crop inventory, crop status assessment, and yield forecasting. Operational cropland mapping must comply with several requirements such as timeliness, accuracy, automation and cost-effectiveness [6]. A critical limitation to achieving timeliness and cost-effectiveness is the availability of *in situ* data to calibrate supervised classifiers. The reliance on within-season *in situ* data or on human interpretation of spectral signatures makes the classification process resource-intensive, time-consuming, and difficult to repeat over space and time. Several strategies have been devised to cope with the limited availability of calibration data such as increasing the amount of field data by identifying homogeneous regions around them, based on aerial photography [7] or by implementing positive and unlabeled learning algorithms [8, 9]. Such one-class classifiers are particularly interesting because the cost of unlabeled samples tends to zero and can thus have a much larger size than the positive sample set. Extracting calibration data from existing land cover maps [10–12] is especially interesting because such maps are already available globally. Other approaches include automatic adaptive signature generalization which derives class signatures from pixels with stable land cover through time [13, 14].

Another challenge for national-scale cropland mapping is to achieve spatial continuity and consistency in the final map. There are two main sources of spatial inconsistencies: heterogeneity in the imagery (different orbits, acquisition dates, cloud/shadow contamination) and within-class spectral variability due to changes in environmental conditions, management decisions and practices. Given the amount of data required to cover large areas, this heterogeneity is likely to propagate in higher level products. Efficient strategies to cope with satellite data heterogeneity and spectral variability are therefore crucial. A first strategy to reduce the spectral variability is to derive temporal or spectral-temporal features from the time series [12, 15–17]. Spectral-temporal features are composites of the spectral reflectances measured at a specific stage in the season. They summarize events that did not necessarily co-occur in composite images. These composites facilitate the discrimination between classes by reducing the within-class heterogeneity and improves the classifier’s extendability [12, 16]. Drawbacks of spectral-temporal features are related the amount of available cloud-free images and their quality. Dense time series are required to be able to extract stable spectral signatures at the key moments in the season. Besides, poor cloud/shadow screening results inevitably to noisy features. Classifiers’ accuracies are affected by the landscape diversity over large areas [18]. In fact, the specific characteristics of the agro-systems to be mapped tend to have a stronger influence on the classification accuracy than the classification methods themselves [19]. Therefore, a second strategy to achieve spatial consistency is to stratify the area of interest, *e.g.*, by agro-environmental conditions, and to calibrate stratum-specific algorithms [20–22]. This kind of local training is generally achieved at a higher processing cost and achieving seamless transitions between strata can be challenging [11].

With the dearth of *in situ* data and the requirement of achieving spatial consistency as a backdrop, we present a method to derive automatically national-scale cropland maps based on multi-sensor Landsat time series and outdated land cover information. Given the merits of the aforementioned strategies for large-scale cropland mapping, we detail i) how consistent spectral-temporal features can be derived from high resolution time series to capture the salient characteristics of cropland, ii) how calibration data can be selected from an outdated land cover map, and iii) how the classifiers’ soft outputs can be used to merge stratum-specific classifications and to improve the majority spatial filter. We tested the method in South Africa to capitalize on a wall-to-wall validation data set, *i.e.*, field boundaries, as well as to assess the method performance in space, document the errors and identify the drivers of accuracy. It is worth noting that we do not present a product but a procedure for mapping national-scale cropland maps in a consistent and reproducible way.

## Study area

**Fig 1:**
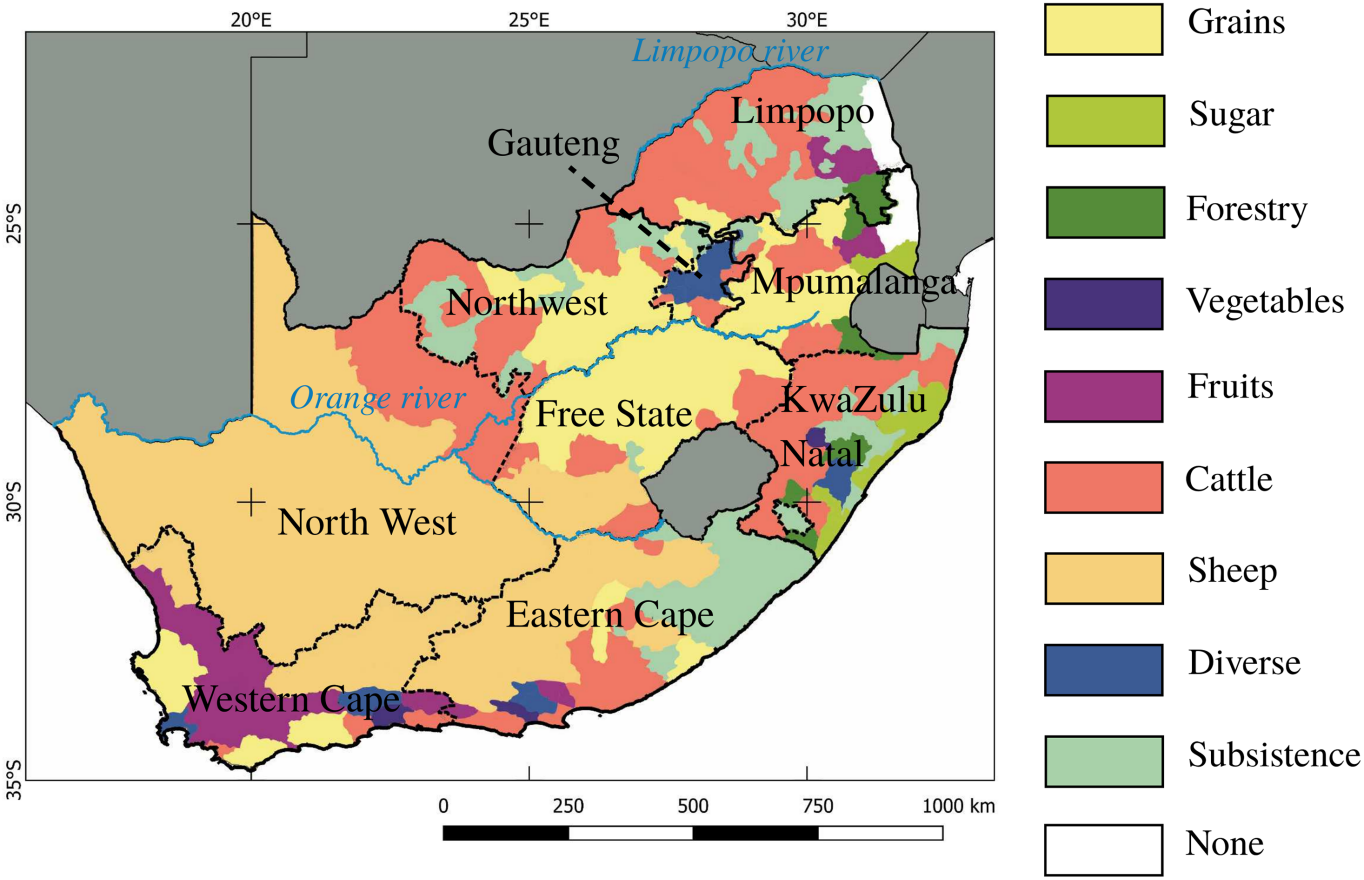
Agricultural regions of South Africa and provincial breakdown. Commercial grain-growing areas are predominantly located in the Western Cape province and in the maize quadrangle (North West and Free State provinces). Subsistence farming mostly occurs in the North West and the Eastern Cape provinces.

South Africa is located at the southern tip of Africa and lies between latitudes 22° and 35°S, and longitudes 16° and 33°E spreading over 1,221,037 km^2^ (Fig 1). The country is divided into nine provinces and has a wide variety of climates ranging from arid to sub-tropical, temperate or Mediterranean. The agricultural economy is a dual, with both well-developed commercial farming and more subsistence-based production in the remote rural areas. The dominant activities include intensive crop production and mixed farming in areas characterized by winter rainfall and high summer rainfall, cattle ranching in the bushveld and sheep farming in the arid regions (Fig 1). About 12% of the territory can be used for crop production but only 22% of this is of high-potential. The main growing regions lie along the more fertile soils of the Western Cape valleys and the KwaZulu-Natal province. Agricultural systems have been primarily developed under arid and semi-arid climatic conditions where droughts are common [23]. Irrigation agriculture is by far the largest consumer of water [5], and is responsible for 30% of the total crop production [23]. The majority of the grain production is irrigated under center-pivot systems, and in many cases based on a double cropping rotation with winter wheat followed by summer maize. Most of the dryland crop production occurs in the semi-arid zones that can be divided into winter and summer rainfall regions.

The largest area of cropland is planted with maize, followed by wheat, and to a lesser extent sugarcane and sunflower [3, 24]. It is estimated that over 8,000 commercial maize producers are responsible for the majority of the South African crop (10.8 Mt of maize produced in 2011/12 on 2.7 million ha of land), while thousands of small-scale producers are responsible for the rest. The “maize quadrangle” in the North West Province and northwestern Free State produces 75% of the country’s maize. Half of the production consists of white maize for human food consumption. Wheat is produced mainly in the winter rainfall areas of the Western Cape and the eastern parts of the Free State (2,0 Mt produced on 0,6 million ha in 2011). Sorghum is cultivated mostly in the drier parts of the summer rainfall areas such as Mpumalanga, the Free State, and Limpopo, especially in shallow and heavy clay soils. Groundnuts are grown mainly in the Free State, North West and the Northern Cape under irrigated or rainfed conditions. Soybeans are mainly cultivated in Mpumalanga, the Free State and KwaZulu-Natal, and are a small but important and growing component of South Africa’s agricultural economy [25, 26]. Sunflower seed is produced in the Free State, North West, on the Mpumalanga highveld and in Limpopo.

### Satellite data pre-processing

All Landsat-5, -7 and -8 data from 2013 to 2015 falling into the area of interest (70 tiles) were acquired and pre-processed following the procedure implemented in [27–29]. Note that four tiles (path/row: 176/77, 175/78, 174/78, 174/79) were discarded because no crop is grown there. Four spectral bands were kept: the red, the near-infrared (NIR), and the two short-wave infrared (SWIR) bands. The blue and green bands were discarded due to their sensitivity to atmospheric effects [30]. We applied a three-step procedure to normalize the radiometry. First, Landsat data were converted to top-of-atmosphere reflectance [31] and then normalized by taking the corresponding MODIS top-of-canopy reflectance data as target [32]. Third, we adjusted cross-track surface anisotropy effects by modeling the Landsat reflectance per spectral band as a function of the viewing angle [32–34]. The above-mentioned processing steps incrementally improved the appearance of the data, providing more spatial coherence and increasing the generalization and internal consistency of the multi-spectral feature space [33].

### Outdated land cover map, validation and ancillary data

The National Land Cover (NLC) 2000 map was generated from Landsat imagery acquired primarily from 2000-2001 [35]. It describes the South African territory with 45 land cover classes and an accuracy of 66%. The minimum mapping unit is 2 ha (approx. 22 Landsat pixels). We translated the NLC-2000 native legend into a simplified nine-class legend: cropland, irrigated cropland, forest, shrubland, grassland, wetland, built-up, bare soil, and water bodies. One can expect that this thematic aggregation led to an increase on accuracy.

For validation, the 2014 national field boundary data set was sourced from the Department of Agriculture, Forestry and Fisheries. It was created by digitizing all fields throughout the country based on 2.5 m resolution, pan-merged SPOT-5 imagery acquired between 2013 and 2015. Field polygons were rasterized at 30 m so that it matched Landsat’s grid, providing a wall-to-wall validation data set rich of >2 billions reference pixels.

Ancillary data were collected in order to assess if local accuracy pattens can be explained by proximity to specific landscape features and/or environmental parameters. Those include the hole-filled Shuttle Radar Topography Mission digital elevation model (90 m, 2003; [36]), the annual precipitation and the mean temperature from the WorldClim database (30 arc seconds, 1960-1990; [37]), the IFPRI-SPAM crop type distribution map (5 arc minutes, 2000; [38]), the irrigated areas coming from the irrigated area map of Africa (250 m, 2010; [39]), the settlement locations from the Global Insight 2012 data set, and the water courses as well as the road network from OpenStreetMap.

## Methods

The method section is structured in four parts. First, we introduce the classification scheme that was developed to update the cropland map of South Africa. The second section introduces the map accuracy assessment using the wall-to-wall validation data set. Then, we detail how we related the spatial patterns of accuracy with explanatory variables. Finally, we present how we assessed the respective importance of the spectral-temporal features in the classification process.

### Classification scheme

**Fig 2:**
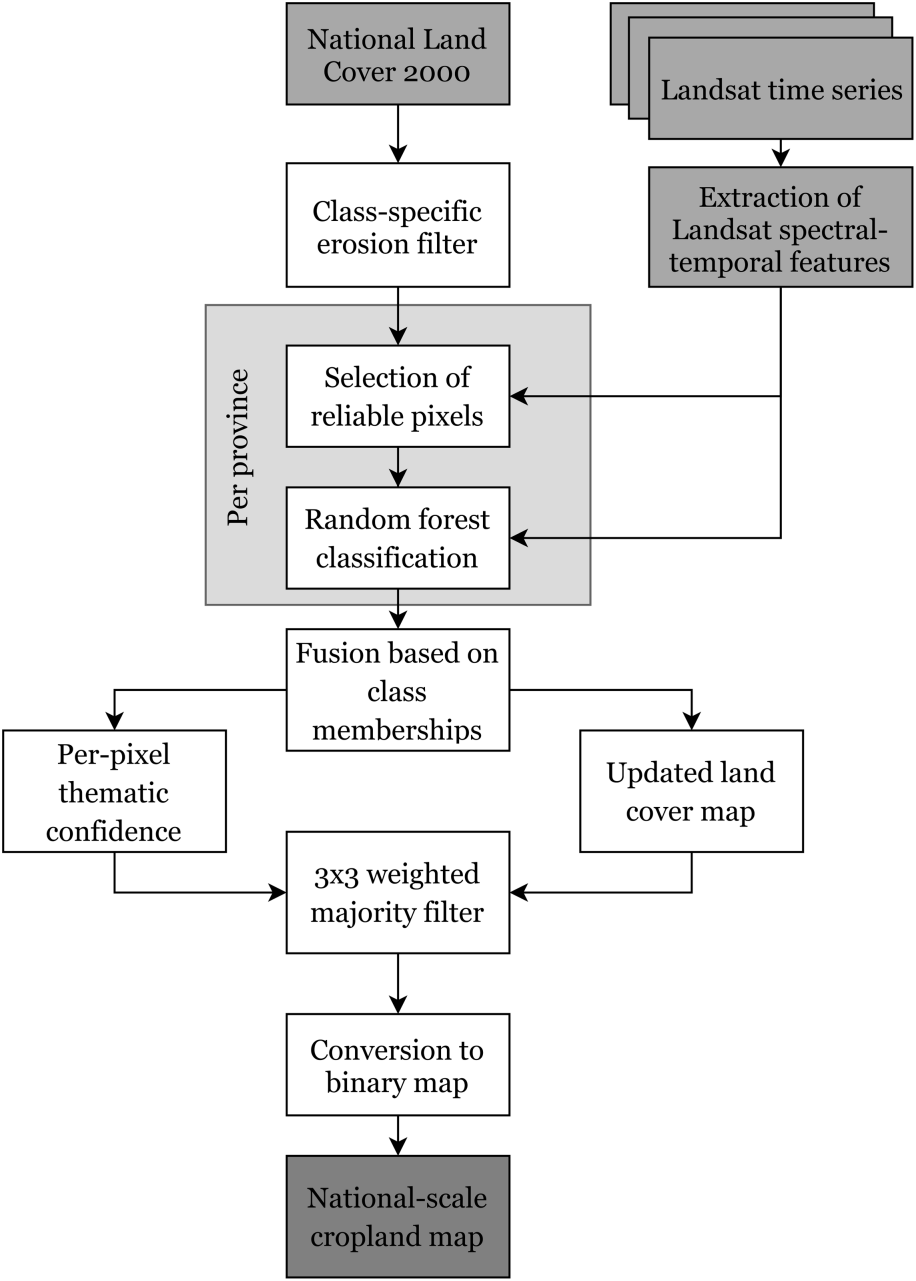
Flowchart of the proposed classification scheme to derive spatially consistent national-scale cropland maps based on outdated land cover information and spectral-temporal features. It highlights the four main steps of the procedure: 1) extraction of the spectral-temporal features, 2) stratum-specific classification, 3) fusion based on class memberships, and 4) speckle removal.

The main originality of the classification scheme is its ability to deal with large territories and thus big volumes of data while remaining fully automated and generic. The classification scheme includes four main steps (Fig 2):

1. extraction of spectral-temporal features from the input time series;
2. stratum-specific classifications based on reliable pixels identified in the outdated land cover map;
3. fusion of the stratum-specific maps based on pixel-level class memberships;
4. speckle removal with a weighted majority filter that takes into pixel-level classification confidence.

Even though the goal is to produce a cropland vs. non-cropland map, the methodology works at the level of land cover classes to enhance between-class discrimination, *e.g.*, between rainfed and irrigated cropland.

#### Extraction of spectral-temporal features

Three spectral-temporal features were derived from all exploitable pixels in the normalized time series, that is, pixels not affected by clouds, cloud shadows, adjacent clouds and quality flags. These features were defined to capture salient crop characteristics:

1. the median reflectance value over the three-year time series (med.red, med.nir, med.swir1, med.swir2);
2. the average reflectance of all pixels belonging to the first decile of stacked NDVI values (minNDVI.red, minNDVI.nir, minNDVI.swir1, minNDVI.swir2);
3. and the average reflectance of all pixels belonging to the last decile of stacked NDVI values (maxNDVI.red, maxNDVI.nir, maxNDVI.swir1, maxNDVI.swir2).

**Fig 3:**
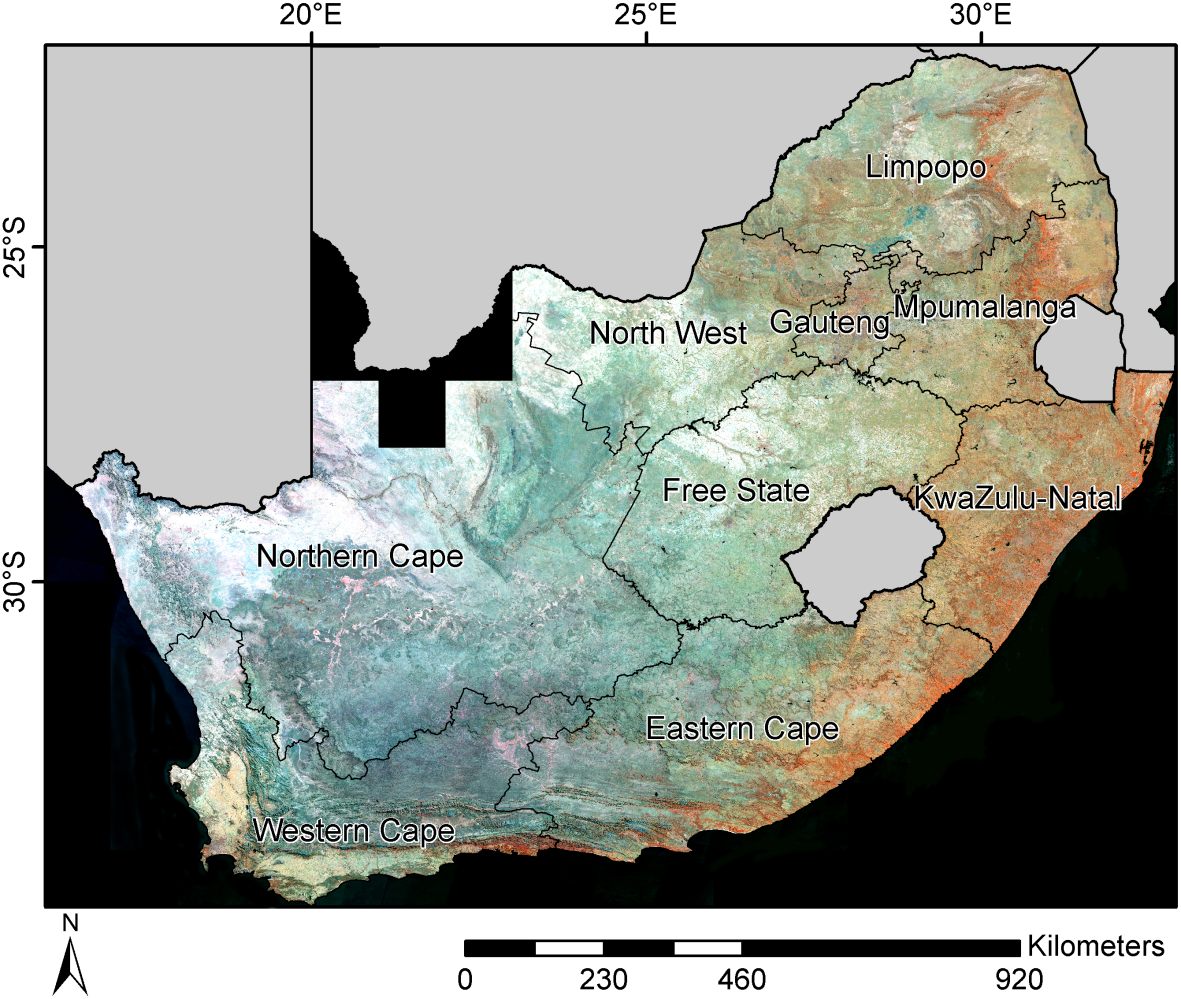
False color composite (minNDVI.red, minNDVI.nir, minNDVI.swir1) of the study area for the years 2013-2015. Forests are shown in red tones, light color depths represent bare soil (including annual cropland), greenish and blueish areas are grassland and shrubland, dark blue pixels correspond to water bodies.

There were thus twelve input features for the classification (three temporal features of four spectral bands each). Fig 3 presents a false color composite (minNDVI.red, minNDVI.nir, minNDVI.swir1) of the study area.

#### Identifying reliable pixels from the outdated land cover map

Outdated land cover maps are subjected to two types of errors: classification errors and land cover changes since the production date. To avoid training a classifier with misclassified pixels, we implemented a procedure to identify “reliable pixels”. Here, “reliable pixels” refers to pixels that are correctly labeled in the outdated land cover map during the period of interest. First, a class-specific erosion filter removed all boundary pixels to account for a potential imperfect co-registration. Second, we selected reliable pixels for training based on an unsupervised clustering of the spectral-temporal features [12]. The underlying assumption is that the cluster purity, *i.e.*, the proportion of pixels of a certain class within a cluster, is a good indicator of the class reliability of the pixels because pixels with similar spectral properties will belong to the same cluster. Therefore, mislabeled pixels are characterized by a low purity because they will likely belong to clusters dominated by another class (their true class), and conversely.

For each stratum, a random sample of 5,000 pixels belonging to a known class, *e.g.*, class *c*, was drawn as well as a sample of 10,000 pixels from all the remaining classes. These two sets were merged and clustered based on their spectral-temporal features using self-organizing maps. The cluster purity was computed and all pixels labeled as *c* and belonging to clusters with a purity of at least 75% were flagged as reliable pixels. Additionally, we filtered out all reliable pixels strongly deviating from the class distribution (95% of confidence interval) in at least one of the four bands of the three features. This reliable pixel selection was repeated for all the classes present in a given stratum. The final training set was constructed by sub-sampling the reliable pixels from all classes in the stratum in order to i) regain the initial class proportions as suggested by [40, 41], and ii) ensure a sample size of 5,000 pixels. The sub-sampling was carried out as to maximize the intra-class dissimilarity using the approach developed by [42]. The class proportions were derived from the NLC-2000 map.

#### Stratum-specific classification to handle the spectral diversity

Because of the vegetation dynamics and the variability of spectral signatures due to environmental gradients and management practices, we stratified South Africa into nine zones according to the province delineation. Province boundaries tend to follow environmental boundaries in some cases and in others, they provide a finer breakdown of the country than the existing environmental stratifications. Then, we calibrated stratum-specific Random Forest (RF) classifiers using the reliable pixels identified previously. RF is a non-parametric, ensemble classifier based on a large set of decision trees and bootstrapping with replacements [43]. As each tree predicts a class, the RF output class is defined by taking the majority vote of all trees. RFs are particularly attractive because they require little guidance for parameterization –especially once the number of trees exceeds 100 [44, 45]. Besides, RFs do not overfit and can handle high-dimensional inputs as well as multicollinearity [44, 46]. Finally, they achieve high robustness to random and systematic label noise up to 25%-30% [47]. This relative insensitivity to noise is especially desirable as undetected mislabeled pixels could occur. Practically, the number of trees was set to 500 and the number of random split variables to the square root of the number of input variables which conforms to the guidelines provided by [44] and [48].

We applied a buffer zone of one third of a degree to minimize boundary artefacts due to the stratum-specific training. In areas where strata overlap, several maps were produced. To integrate them, we relied on per-pixel class memberships, *i.e.*, the vote distribution of the trees between the input classes: *p* = {*p*_1_, *p*_2_, …, *p*_*i*_, …, *p*_*n*_} where *p*_*i*_ is the estimated membership of a given pixel to class *i*, and *n* the number of classes. We fused the class memberships using a geometric mean operator [49] and attributed the final class following the maximum likelihood principle. In non-overlapping areas, fusion was not necessary and the final class was extracted from to the only stratum-specific classification available.

#### Post-classification filtering

Post-processing methods such as spatial filters are often applied to classified maps for speckle removal. This can be an important step in improving their quality [50–54]. An oft-used spatial filter is the majority filter which replaces isolated pixels by the majority class in the moving spatial window.

$$\begin{array}{c} M\left(s\right)={\text{argmax}}_{i}\sum_{j}^{n}I(h_{j}\left(s\right)=i) \end{array}$$ (1)

where *h*_*j*_ are votes, *I*(⋅) is an indicator function and *n* is the number of classes and *M*(*s*) is the final label for a given spatial window *s*. However, conventional majority filtering results in inevitable information loss and classification errors at boundary are not dealt with effectively [55, 56]. The main reason is that this type of spatial filtering applies arbitrary weights to all locations and it could be enhanced by accounting for pixel-level classification confidence. Pixel-level classification confidence measures can be derived from the soft outputs of the classifiers. Rooted in information theory, the Equivalent Reference Probability (ERP; [57]) is particularly interesting because it accounts for the full set of probabilities while remaining consistent with the maximum probability. Pixels classified with high confidence have an ERP close to unity. A modified version of the filter is proposed in order to account for pixel-level classification confidence information into:

$$\begin{array}{c} M_{w}\left(s\right)={\text{argmax}}_{i}\sum_{j}^{n}\omega_{j}I\left(h_{j}\left(s\right)=i\right) \end{array}$$ (2)

where *ω*_*j*_ is the weight. The weights were obtained by normalizing the ERP values within the moving windows so that they sum to 1. We implemented the weighted majority filter with a moving window of 3x3 pixels and reclassified the land cover map into a binary cropland/non-cropland map after filtering. For completeness, we compared the weighted majority filter with conventional majority filter and without spatial filtering.

### Evaluation of the classification accuracy

The classified pixels were compared to the pixels from the wall-to-wall validation samples. Accuracy measures such as the overall accuracy (OA) [58] as well as the F-scores for the cropland class (FS_*C*_), and the non-cropland class (FS_*NC*_) [59] were derived from the confusion matrix. The F-score is a class-specific accuracy metric mathematically defined as the harmonic mean of the users’ and producers’ accuracies of the class being evaluated. The standard error of the overall accuracy estimates is also provided.

Map accuracy is known to vary in space [21, 60–62]. Capitalizing on the wall-to-wall data set, local variations of the accuracy measures were characterized by constraining geographically the reference data used for validation [60]. Following a regular grid of points spaced 40 km apart, local accuracy measures were computed by considering all pixels falling within a 90x90 km^2^ spatial window. We interpolated the accuracy measures with an inverse distance weighting approach.

### Explanatory variables of the classification accuracy

The potential of several variables to explain and predict the spatial variability of classification accuracy was evaluated. Together, these variables describe landscape and climate characteristics as well as the physical, environmental and agricultural management conditions. They were selected because of their potential to describe different cropping systems, *e.g.*, irrigation is highly likely to occur in areas close to rivers and intensive fields generally occur in accessible areas. By extension, different cropping practices have different spectral signatures which can be recognized with different success rates. They can be divided into three groups (Table 1):

1. *Site-specific characteristics* describe the physical and climate conditions. They also characterize local cropping practices such as crop diversity and irrigation. The crop diversity layer was interpolated to the whole country using a minimum distance algorithm.
2. *Density characteristics* relate to the intensiveness of agriculture (field density) and urbanization (road density) as well as to the potential for irrigation. They were computed using a kernel density approach that fitted a smoothly tapered surface to points or lines. The search radius is computed specifically to the input data set using a spatial variant of Silverman’s Rule of thumb.
3. *Proximity characteristics* including distance to roads, rivers, settlements or agriculture were also calculated. Factor maps depicting distances were calculated as the Euclidean distance to the nearest feature.

**Table 1:** Potential explanatory variables of accuracy. These variables are of three types: site-specific characteristics, density characteristics and proximity characteristics.

Variable	Description
*Site-specific characteristics*	
Elevation	Elevation (m)
Slope	Slope (%)
Mean annual rainfall	Average annual temperature (mm)
Mean annual temperature	Average annual temperature (°C)
Crop diversity	Average number of crops (number of crops/km^2^)
Irrigation proportion	Proportion of crops under irrigation [%]
*Density characteristics*	
River density	Density of rivers (km/km^2^)
Road density	Density of roads (km/km^2^)
Field density	Density of fields (number of fields/km^2^)
*Proximity characteristics*	
Distance to roads	Euclidean distance to roads (km)
Distance to rivers	Euclidean distance to the nearest river (km)
Distance to settlements	Euclidean distance to the nearest settlement (km)
Distance to fields	Euclidean distance to nearest field centroid (km)

We evaluated the degree of association of the explanatory variables with the overall accuracy and the F-scores with multivariate adaptive regression splines (MARS) [63]. MARS is a non-parametric statistical method relying on a divide and conquer strategy that portions training data sets into separate piece-wise linear segments (splines) of differing gradients (slope).

We calibrated one MARS model per accuracy measure. The importance scores of a predictor variable were calculated by refitting the model after dropping all terms involving the variable in question and tracking the corresponding reduction in goodness-of-fit. The best predictor variable degraded the model fit the most, and conversely [64]. Three statistics of the MARS model express the variable importance: i) the generalized cross-validation statistic (GCV) [65], ii) the residual sum of squares (RSS) and the number of times that each variable is involved in an optimal subset, iii) the number of times that each variable is involved in a subset (in the final, pruned model). We extracted the accuracy measures and the predictor variables at 4,000 locations following a systematic sampling scheme. Two thirds were used for calibration and one third was set aside for independent validation.

### Remote sensing features of importance

RF provides measures of the feature importance in the classification process such as the Gini index [43]. Gini indices were thus analyzed to identify the influential spectral and temporal features for each stratum. A Friedman test [66] and a post-hoc Nemenyi test [67] were performed to determine if the ranking of the features was stratum-specific.

## Results

### Visual assessment

**Fig 4:**
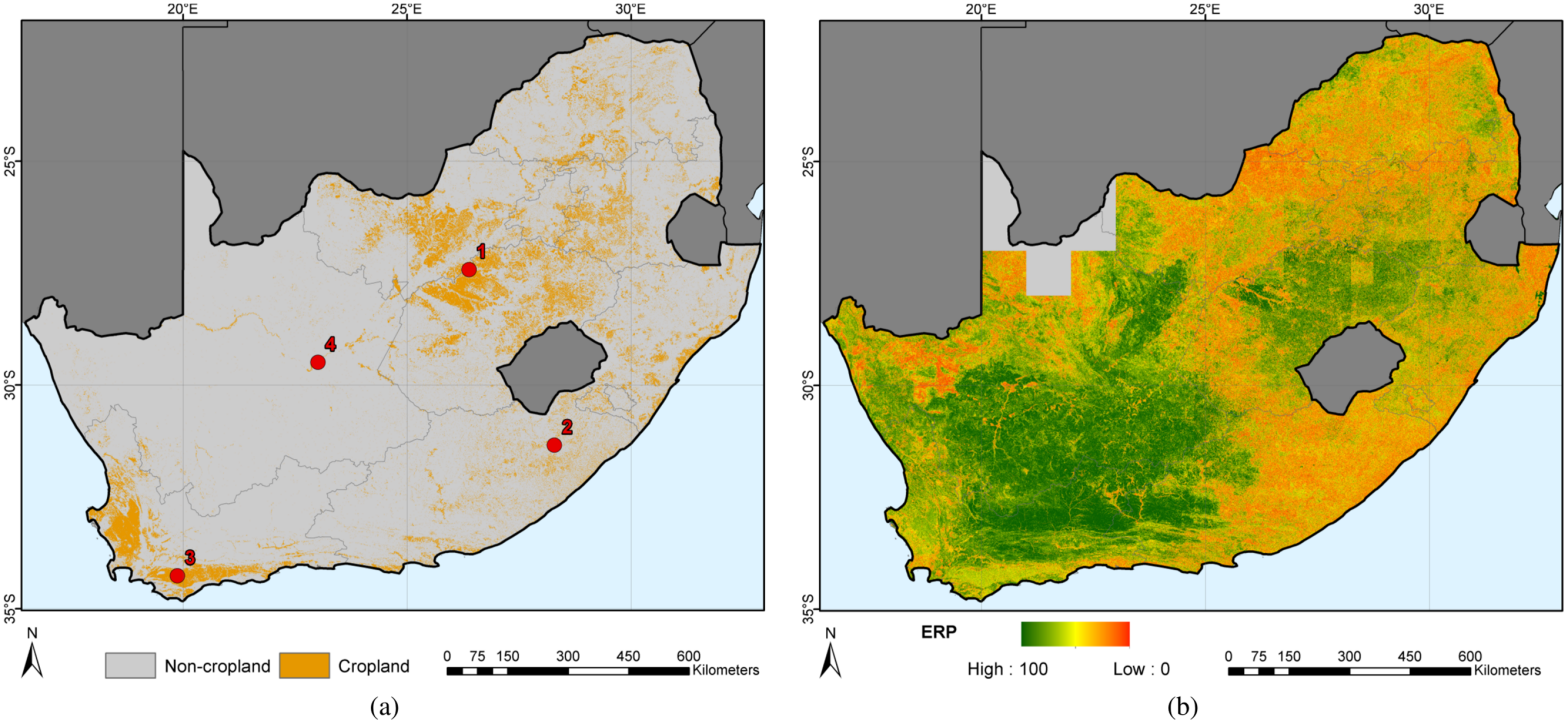
Updated cropland map of South Africa for the 2013-2015 period. Fig 4a) illustrates the national-scale cropland map and Fig 4b) shows the corresponding pixel-level confidence map. The red points are the locations of the four zooms of Fig 5.

**Fig 5:**
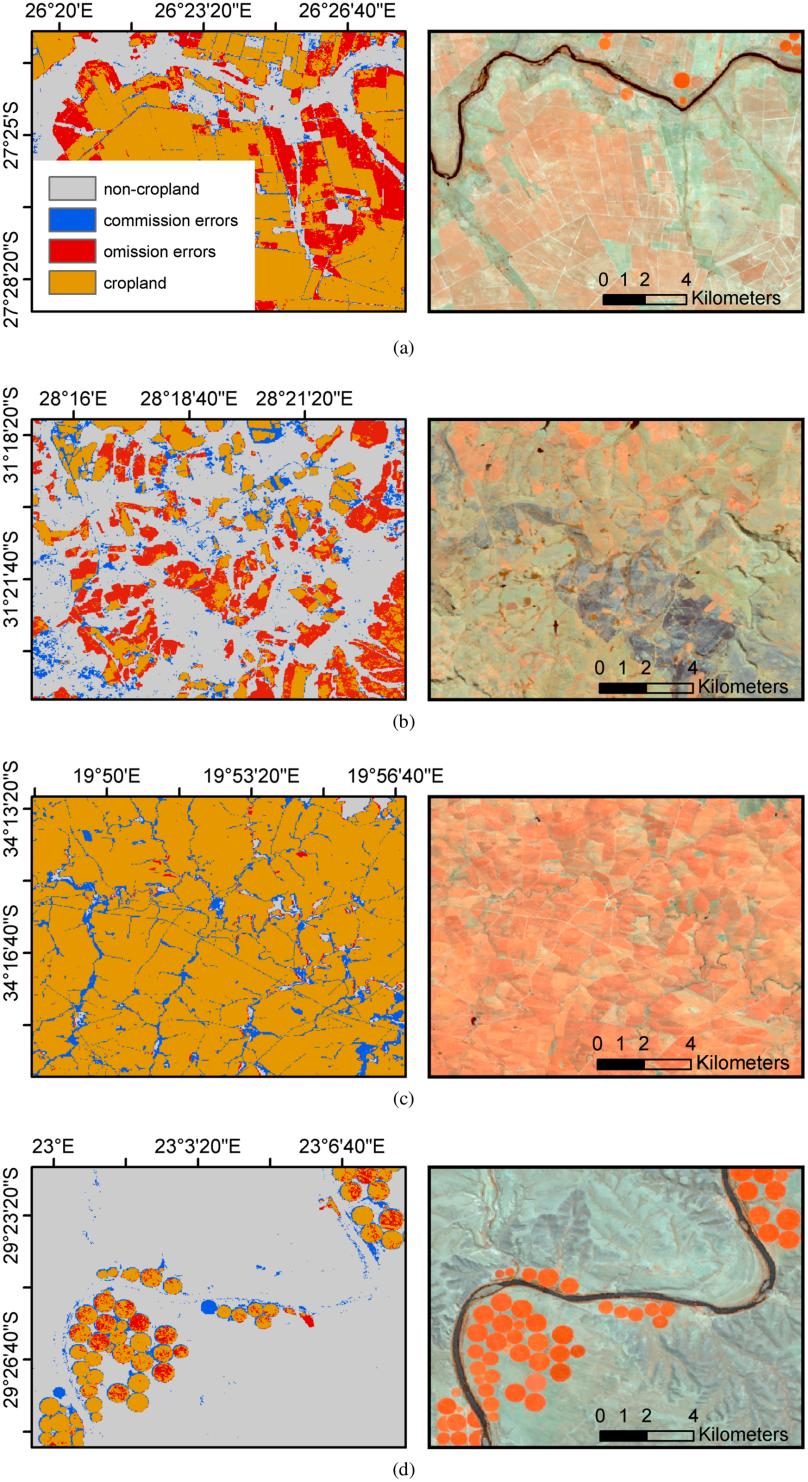
Selected zooms over four contrasted sites at the 1:200,000 scale. Left-hand side images for the four subsets provide a synoptic view of South African Cropland as well as its accuracy since areas in red represents omission errors, and areas in blue commission errors. Right-hand side images are false color composites of the maximum NDVI Landsat feature (maxNDVI.swir1, maxNDVI.nir, maxNDVI.red).

The updated national-scale South African cropland map depicts the typical patterns of the country’s cropland, *i.e.*, two intensively-cropped areas: an L-shaped area in the Western Cape and another centered on the Free State (Fig 4). Two large patches of high pixel-level confidence are visible: the first one includes most cropped areas in the Free State, the second incorporates most of the Western and Northern Cape provinces. Overall, a good level of spatial consistency is achieved in both maps. The four red points marked the locations of the four zooms provided in Fig 5. Each subset illustrates a different landscape ranging from intensive large fields in the western Free State and in the Western Cape provinces (Fig 5a) and 5c)) to fields under pivot irrigation in the Northern Cape province (Fig 5d) and smaller fields of the Eastern Cape (Fig 5c). The left-hand side image provides a synoptic view of the classification and of its accuracy since areas in red represents omission errors, and areas in blue commission errors. The right-hand side image is a false color composite of the maxNDVI feature (maxNDVI.swir1, maxNDVI.nir, maxNDVI.red). As this composite corresponds to a maximum NDVI composite, *i.e.*, illustrating the maximum photosynthetic activity of the period of interest, reddish areas are associated with high photosynthetic activity where blueish areas correspond low photosynthetic activity (bare soil, fallow). Dark blue colors are burned areas.

The analysis shown in Fig 5 yields the following observations. Besides obvious misclassification errors, omission errors (in red) seem to occur in areas that were previously cropped or under fallow during the period of interest (see the large red patches in Fig 5a) and 5c) for instance). Commission errors (in blue) with natural vegetation occurs along the river which highlights the challenge of discriminating irrigated crops from riparian vegetation. They also tend to consistently affect pixels close to field boundaries and between fields. This might be related to the similarities between the spectral-temporal signatures of cropland and the surrounding grassland. Yet, this is insufficient to explain the inability to separate road pixels (Fig 5c)). Commission might thus also be attributed to the net point spread function of the sensor for two reasons. First, it decreases the separability between pixels by mixing their signal [68, 69]. Second, it was neglected when resampling the field boundaries to Landsat’s spatial resolution, thereby introducing a pessimistic bias on the accuracy estimation. Finally, some commission errors are due to pixels that are cropped on the composite but not digitized in field boundary dataset. The multi-year component of both the features and the validation data set as well as the imperfect co-registration of the imagery with the wall-to-wall validation data are sources of a pessimistic bias in the accuracy estimation.

### Map accuracy assessment

The accuracy of the national-scale cropland map was 92.0% (Table 2). Regarding per-class accuracies, the F-scores reached 63.3% for the cropland class and 95.3% for the non-cropland class. The weighted majority filtering slightly improved the accuracy compared to conventional majority filtering or to no filtering. Due to the high number of pixels used for the computation of the accuracy measures (>2 billion pixels), the standard deviation of the overall accuracy is very small. Thus, all differences between accuracies can be considered as highly statistically significant. Bearing in mind the limitations of the validation data set due to the imperfect co-registration, we re-computed the accuracy after discarding all boundary pixels. The overall accuracy reached 95.7% and the F-score for the non-cropland class reached 97.7%. The most significant increase was observed for the F-score of the cropland class (71.5%; +7.3%). Further, we calculated Mann-Whitney-Wilcoxon tests to assess if the population of pixel-level confidence differed for well-classified and misclassified cropland pixels. For each stratum, well-classified cropland pixels tend to have a higher ERP value, *i.e.*, a higher classification confidence, than misclassified pixels at the .05 significance level.

**Table 2:** Accuracy measures for different post-filtering scenarios. The overall accuracy (OA) is given with the standard deviation (SD) of its estimation. Weighted majority filter performs better than the conventional majority filter and no filtering. Discarding edge pixels increased the accuracy, highlighting the difficulty to classify boundary (mixed) pixels and a less than perfect co-registration between the field boundary data set and the Landsat data.

Post-processing	OA +/- SD [%]	FS_*C*_ [%]	FS_*NC*_ [%]
Weighted majority filter	92.0 ± 4.20e-9	64.2	95.5
Weighted majority filter + erosion of edge pixels	95.7 ± 4.20e-9	71.5	97.7
None	91.7 ± 4.34e-9	63.3	95.3
Majority filter	91.9 ± 4.23e-9	63.9	95.5

**Fig 6:**
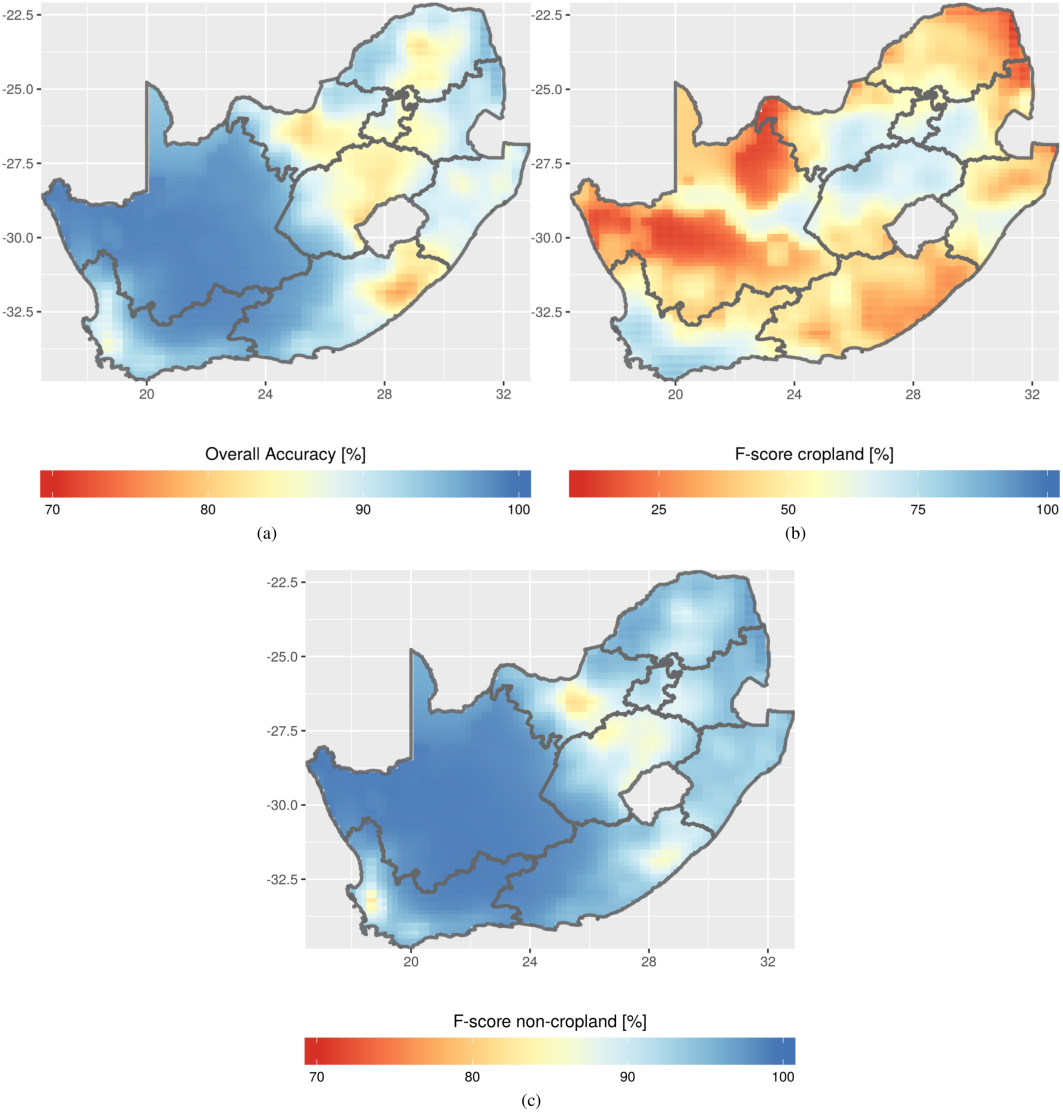
Spatially constrained accuracy assessment for the three accuracy measures. Cold spots of overall accuracy and F-score for the non-cropland class occur where the crop proportion is low. Hot spots of F-score for the cropland class are in intensive grain-growing regions and irrigated areas. Note the different color scales.

Local measures of the thematic accuracy were derived from spatially constrained confusion matrices to illustrate the local variation of accuracy in the map (Fig 6; S1 File). The overall accuracy and the F-score for the non-crop class follow generally the same spatial patterns. This behavior was expected given prevalence of non-cropland pixels. Cold spots of those measures coincide with areas where the crop proportion increases, *i.e.*, where the probability of misclassification is likely. Regarding the accuracy of the cropland class, two hot spots of accuracy are visible and coincide with the two intensive grain-growing areas of the country, one in the Western Cape province and the other in the maize quadrangle (see Fig 1). Irrigated areas along the Orange river (from the center of the country to the Namibian border) are also mapped with high accuracy. Cold spots of accuracy occur in landscapes with low cropland proportion dominated by smallholder farming.

### Drivers of accuracy

Table 3 presents the generalized cross-validation estimate of error of the MARS models, the residual sums of squares, and number of times that each predictor variable is involved in a subset of a pruned model (S2 File). A very good fit was achieved for all three models, indicating a high prediction power of the variables. The Pearson’s correlation coefficients between the predicted and the observed accuracies were superior to 0.84. Besides, the root mean square errors were particularly low: 0.015, 0.02, 0.085 for the models based on the overall accuracy, the FS_*NC*_, and the FS_*C*_, respectively. The overall accuracy tends to decrease as the annual precipitation, the distance to fields, the field density and settlement density increase. For the non-cropland class, the F-score decreases as the altitude, temperature, field density and settlement density increase. Besides, it diminishes as the precipitation increases until it reaches 600 mm, then the trend is inverted. For the cropland class, the F-score increases with field density but decreases as annual precipitation and settlement density increase. The analysis of the variables driving the accuracy converges with the previous spatial analysis and suggests that the method performed best in farming systems with a high productivity.

**Table 3:** Explanatory variable of accuracy derived from the MARS models. Three parameters are provided: the generalized cross-validation (GCV) estimate of error, the residual sums of squares (RSS) as terms are added, and number of times that each variable is involved in a subset in the final, pruned model (nsubsets).

**Overall accuracy**			
	**nsubsets**	**GCV**	**RSS**
Annual precipitation	23	100.0	100.0
Field density	22	44.4	44.9
Settlement density	21	35.2	35.8
Elevation	19	29.3	30.0
Distance to fields	19	29.3	30.0
Annual temperature	18	27.5	28.2
Distance to rivers	17	22.1	23.0
Crop diversity	12	12.3	13.4
Slope	7	6.5	7.8
		0.0004	1.2
**F-score cropland**			
	**nsubsets**	**GCV**	**RSS**
Field density	23	100.0	100.0
Settlement density	22	76.5	76.9
Distance to rivers	21	58.2	58.9
Annual temperature	20	48.0	48.9
Annual precipitation	19	40.9	42.0
Crop diversity	19	40.9	42.0
Elevation	17	32.3	33.7
		0.0074	21.9
**F-score non-cropland**			
	**nsubsets**	**GCV**	**RSS**
Field density	26	100.0	100.0
Annual precipitation	25	53.4	53.9
Annual temperature	24	43.0	43.6
Elevation	23	40.2	40.8
Distance to fields	22	36.4	37.1
Slope	22	36.4	37.1
Settlement density	20	31.1	31.8
Road density	16	23.7	24.5
Irrigation proportion	10	17.6	18.2
		0.0002	0.714

### Spectral-temporal features of importance

**Fig 7:**
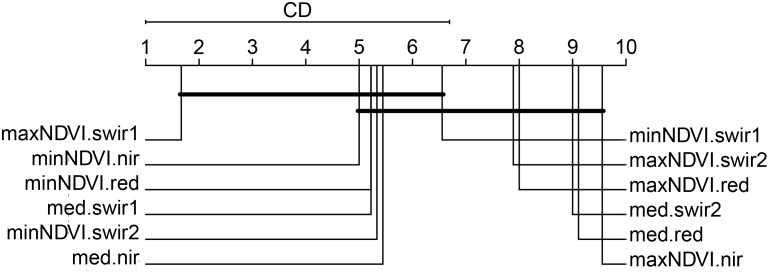
Critical distance diagram. The top line in the diagram is the axis along which the average rank of each spectral-temporal feature is plotted, from the lowest ranks (most important) on the left to the highest ranks (least important) on the right. Groups of features that are not statistically different from one another are connected. The critical difference (CD) is shown above the graph.

The variable importance was quantified with the Mean Decrease Gini at the province level (S3 File). First, a Friedman test was performed and lead to the rejection of the hypothesis that all features are equivalent at the .05 significance level (Friedman’s chi-squared = 40.111; *p*-value = <0.001). We applied a post-hoc Nemenyi test for a pairwise comparison of the average ranks of all 12 features. According to this test, the feature importance is significantly different if the average ranks differ by at least the critical difference CD = 5.6941. As proposed by [70], the critical distance diagram summarizes these comparisons (Fig 7). A connecting line between features means that the null hypothesis of them being significantly different from one another could not be rejected. Despite the inter-province variability, three groups can be identified. The first is constituted of the first SWIR band of maxNDVI which seems important regardless of the province. A second group stands out with minNDVI.nir, minNDVI.red, med.red, minNDVI.swir2, med.nir. Finally, the third group is formed by the less important features –maxNDVI.swir2, maxNDVI.red, med.swir2, med.red, maxNDVI.nir.

## Discussion

In order to provide up-to-date national-scale cropland information in the absence of within-season ground truth data, we developed a strategy to select reliable calibration pixels from an outdated land cover map based on their spectral signatures. To ensure spatial consistency in the map, we derived seamless spectral-temporal features that capture the salient characteristics of crops from normalized Landsat time series. We also stratified the country to reduce the within-class variability of the spectral-temporal signature. The class memberships derived from the random forest algorithm were instrumental in combining stratum-specific classification as well as to calibrate the weighted majority filter. We applied this method in South Africa and the overall accuracy of the map yielded 92% with some local variations (86 to 99% at the stratum-level). Such accuracy levels are comparable to those attained by similar studies [71–74]. [72] concluded that spectral-temporal features were found instrumental to reach >90% accuracy and to minimize outliers. Frequent errors are observed due to spectral confusions with similar classes such as grassland [12, 75] and pasture [73, 74]. Yet, these works have in common that the calibration data was collected by photo-interpretation or *in situ* and that they covered significantly smaller extents. Thus, extracting data from existing maps appear as a competitive option for cropland mapping, especially where ground truth data is lacking and cannot be collected.

We observed marked accuracy patterns across the country. The overall accuracy and the F-scores for the non-cropland class were high across the country except in areas with high cropland proportions. F-scores for the cropland class were the highest in the two intensive agricultural areas (Western Cape province and maize quadrangle) as well as along the Orange river. Annual precipitation, field density, and settlement density were found to be important drivers of accuracy. [76] already noted a correlation of larger field size with higher classification accuracy and potentially, with a broader range of significant variables including the proportion of crop in the scene, crop diversity, soil order and drainage class, percent slope, maximum yield, geographic location, weather, and crop development stage. In a Sudano-Sahelian landscape, [21] explained 41% of the variance of the classification with eight explanatory variables describing the landscape, the site location and the data availability.

Both the spatially explicit validation and the explanatory variables of accuracy pointed out to the fact that smallholder farming systems were the least accurately mapped. Smallholder farming systems have reportedly been noted as challenging to map with accuracy [75]. This is well illustrated by [77] who concluded that a classification method that yielded good results in commercial farming systems could not deal with smallholder systems due to the small field size. Similarly, accuracies obtained by [78] were always higher than 80% for sites of intensive farming and stalled at around 50% for sites dominated by smallholder agriculture. While dependable information on commercial farming systems is critical to reduce uncertainty in the global commodity markets, traditional smallholder farming systems dominate the savanna range countries of sub-Saharan Africa and provide the foundation for the region’s food security [79]. More generally, estimates suggest that in the rural areas of developing countries around half of the population is smallholder farmers with up to three hectares of cropland [80]. For complex landscapes, methods could benefit from the addition of very high spatial resolution imagery [81] with good temporal information [82] or from any other satellite-derived environmental information, such as elevation data [83]. The spatial resolution of Landsat time series limits without any doubt the accuracy with which fields can be resolved because of the mixed pixels and the resolution bias they introduce [84]. In fact, [85] showed that for area estimates, based on pixel counting from Landsat data, could not reach a 10% accuracy target in most South African landscapes, expect the two intensive grain-growing regions. This highlights that the achievable accuracy is strongly constrained by the resolution and the fragmentation of the cropland.

Spatial variations of accuracy could be mapped thanks to the field boundary data. In general, validation data are not available in such abundance preventing the implementation of local accuracy assessment. In those cases, pixel-level uncertainty information received growing interest in the remote sensing community [12, 86, 87] because they can inform the users of the map of the spatial variations of the quality. There are strong links between accuracy and per-pixel confidence [15, 88]; for instance, we found that well-classified cropland pixels have on average a statistically confidence value. Nonetheless, the information provided by confidence measures such as the equivalent reference probability remains complementary to accuracy measures.

The feature importance analysis underlined the importance of the SWIR band for crop classification as already reported [21, 89–93]. The importance of the SWIR band ought to be related to a differential leaf water content between crops and natural vegetation [94], especially in irrigated areas as well as to its specific links with canopy structure and crop residues. From a temporal perspective, three out of the top five spectral-temporal features come from the minimum NDVI which confirms that cropland is most separable when the soil is bare or prepared for sowing [12, 17].

The availability of 10-m satellite data such as Sentinel-1 and Sentinel-2 provides positive perspectives of improvement to increase the accuracy of the proposed classification scheme, especially in smallholder farming systems where a higher spatial resolution is required. A higher density of images along the growing season would also allow to move toward annual cropland mapping, thereby reducing confusions due to land cover and land use change. The red-edge bands available with Sentinel-2 could be instrumental to enhance discrimination with grassland and wetland vegetation [95]. Besides more accurate and up-to-date land cover data could be used instead of the NLC-2000, *e.g.*, GlobLand 30 [96], and ancillary data geographic databases such as OpenStreetMap could also be included. Advanced filtering method of the reference land cover map such as that proposed by [11] should be tested. Other uses of per-pixel class membership or confidence information should be investigated, *e.g.*, in a scheme to fuse the outcomes of multiple classifiers [97–99] or in an iterative classification process.

## Conclusions

We presented a fully automated methodology to map the cropland extent over large areas based on outdated land cover information and high resolution spectral-temporal features. Special attention was dedicated to ensuring spatial consistency and coherence in the map. We first normalized the Landsat time series and derived spectral-temporal features to obtain seamless input data. Second, the spatial variability of the class signatures was reduced by stratifying the country into homogeneous strata that were classified independently. Applying buffers around each stratum limited artefacts at their boundaries. The stratum-specific maps where finally fused based on pixel-level class membership values and a weighted a weighted majority filter based on pixel-level classification confidence further removed speckle. The classification scheme was demonstrated over South Africa –a country of 1.221 million km^2^– with multi-sensor Landsat-5, -7 and -8 imagery. The cropland map was provided with a confidence map which gives information at the pixel level about the expected thematic quality. Based on a wall-to-wall validation data set, the overall accuracy reached 92%. Imperfect co-registration and land use land cover changes during the period of interest are additional sources of discrepancies between the validation data and the imagery, resulting in a pessimistic accuracy estimation. This level of accuracy is close to what most state-of-the-art methods can achieve when ground truth data are available and could be improved by using more up-to-date input maps and more ancillary data. Smallholder farming systems were more challenging to map than the intensive producing areas because of their higher fragmentation and diversity. Dedicated approaches in terms of methodology and Earth Observation data, *e.g.*, <10-m time series, should be investigated to lower confusions in those complex farming systems. Overall, the method shows potential to regularly produce consistent national-scale cropland maps where *in situ* data are not available.

## Supporting information

S1 File Accuracy assessment with spatially-constrained confusion matrices. The overall accuracy, the user’s and producer’s accuracies, the F-scores, and the uncertainty observed at 1181 locations.
(CSV)

S2 File Explanatory variables of accuracy. The file provides the overall accuracy, the F-score for the cropland and the non-cropland classes, the elevation, the slope, the mean annual rainfall, the mean annual temperature, the crop diversity, the irrigation proportion, the river density, the road density, the field density, the distance to roads, the distance to rivers, the distance to settlements, and the distance to fields at 4680 locations.
(CSV)

S3 File Gini analysis per province. The file shows the Gini index fall all bands of the spectral-temporal features for the nine provinces.
(CSV)
